# Factors Influencing the Concentrations of the Large Neutral Amino Acids in the Brain and in the CSF of Dogs After Portacaval Anastomosis

**DOI:** 10.1155/1991/53689

**Published:** 1991

**Authors:** Torbjörn Jonung, Adel Ramzy, Per Herlin, William T. Chance, J. Howard James, Josef E. Fischer

**Affiliations:** 1 Department of Surgery University of Cincinnati Medical Center Cincinnati Ohio USA; 2 Department of Surgery Lund University Lund 22185 Sweden

## Abstract

Portal-systemic shunting of blood is associated with hyperammonemia, an increased glutamine concentration in brain, an altered plasma neutral amino acid pattern, and high levels of several of the large neutral amino acids in brain. Since some of these amino acids are precursors for neurotransmitters and for other potentially neuroactive substances, high CNS levels of these amino acids may contribute to the development of encephalopathy. In order to determine the relative importance of changes in brain glutamine levels and changes in competition among the neutral amino acids for blood-brain transport, we measured the concentrations of the large neutral amino acids in plasma, cisternal cerebrospinal fluid and in brain tissue from various regions of dogs after end-to-side portacaval shunt. Although the changes in CSF amino acid levels correlated partially with altered amino acid plasma competitor ratios, better correlations were observed with the elevation of CSF glutamine. These results suggest a model of blood-brain amino acid transport in which a high level of glutamine in brain extracellular fluid competes with other neutral amino acids for efflux from brain, thus raising brain amino acid levels after portalsystemic shunting


*HPB Surgery*, 1991, Vol. 4, pp 299–312
Reprints available directly from the publisher
Photocopying permitted by license only

© 1991 Harwood Academic Publishers GmbH
Printed in the United Kingdom

### FACTORS INFLUENCING THE CONCENTRATIONS OF THE LARGE NEUTRAL AMINO ACIDS IN THE BRAIN AND IN THE CSF OF DOGS AFTER PORTACAVAL ANASTOMOSIS

TORBJÖRN JONUNG, ADEL RAMZY, PER HERLIN, WILLIAM T. CHANCE, J. HOWARD JAMES and JOSEF E. FISCHER
*Department of Surgery, University of Cincinnati Medical Center, Cincinnati, Ohio, USA*

*(Received 23 April 1991)*

Portal-systemic shunting of blood is associated with hyperammonemia, an increased glutamine concentration in brain, an altered plasma neutral amino acid pattern, and high levels of several of the large neutral amino acids in brain. Since some of these amino acids are precursors for neurotransmitters and for other potentially neuroactive substances, high CNS levels of these amino acids may contribute to the development of encephalopathy. In order to determine the relative importance of changes in brain glutamine levels and changes in competition among the neutral amino acids for blood-brain transport, we measured the concentrations of the large neutral amino acids in plasma, cisternal cerebrospinal fluid and in brain tissue from various regions of dogs after end-to-side portacaval shunt. Although the changes in CSF amino acid levels correlated partially with altered amino acid plasma competitor ratios, better correlations were observed with the elevation of CSF glutamine. These results suggest a model of blood-brain amino acid transport in which a high level of glutamine in brain extracellular fluid competes with other neutral amino acids for efflux from brain, thus raising brain amino acid levels after portal-systemic shunting.

KEY WORDS: Portacaval anastomosis, portal-systemic, encephalopathy, brain and CSF glutamine, large neutral amino acids, blood-brain barrier

### INTRODUCTION

One of the interesting problems in the study of hyperammonemia, liver failure and portal-systemic encephalopathy is the relationship of changes in the brain of the neurotransmitter-precursor amino acids to the development of encephalopathy. The amino acids of particular interest are tyrosine, phenylalanine and tryptophan, since they are involved in the synthesis in the brain of catecholamines, serotonin and of potential false transmitters such as octopamine or tryptamine[7,5]. These amino acids, together with leucine, isoleucine, valine, methionine, threonine and histidine, comprise the group of large neutral amino acids (LNAA) which share a common blood-brain transport system. In both humans and experimental animals with hepatic failure, many LNAA accumulate in the CNS to a greater extent than

Address correspondence to: Torbjörn Jonung M.D., Department of Surgery, Lund University, 22185 Lund, Sweden the changes in plasma concentrations of those amino acids would seem to predict[18,11,15]. The reason for this "excess" accumulation of LNAA in the CNS of patients or experimental animals with portal-systemic shunting and hyperammonemia has not been completely explained, although data have been presented which point to a role for elevated glutamine concentrations in the CNS in altering LNAA transport at the blood-brain barrier[1,12,13]. In the following study in dogs, we compared the changes after portacaval anastomosis (PCA) in LNAA concentrations in plasma, cisternal CSF and in brain tissue. The latter was obtained only at sacrifice, but the plasma and CSF were sampled sequentially after PCA in order to follow changes over time. The results point to a significant role of elevated brain GLN as a competitor for efflux of the LNAA from the brain to the blood.

### MATERIAL AND METHODS

Sixteen conditioned dogs weighing 9.5–23.9 kg were studied. Twelve dogs were anesthetized with sodium pentobarbital and subjected to an end-to-side portacaval shunt. The anastomosis was done with 5-0 Prolene sutures using the portal vein stump above the last splanchnic branch. After the operation 5% dextrose and saline solution were infused for 24 hours and penicillin and streptomycin were given. All dogs were allowed chow ad libitium. Weight, but not food intake, was monitored. The remaining four unoperated dogs served as controls.

From previous experience in this laboratory[24] with this experimental model, we anticipated that the development of encephalopathy in each dog would be difficult to predict. We intended to sacrifice similar numbers of dogs in Stage I (mild), Stage II (moderate) and Stage III (severe) encephalopathy. Therefore, starting from the second postoperative day two observers independently rated the dogs for encephalopathy. The parameters used for this evaluation were spontaneous activity, ataxia, salivation, ease of arousal, response to foot pinching and abnormalities of gait. The animals were clinically graded as follows:

Grade O: Indistinguishable from normal (no animals were sacrificed in this condition).
Grade I: Less lively, with delayed responses.
Grade II: Hypersalivation; hyperactive movements; walking close to the walls with an unsteady gait.
Grade III: Excessive salivation; sleeping most of the time; when forced to walk, walking "into" walls; standing with great difficulty; very slow response to foot-pinch; coma.

Samples were obtained as follows: Venous blood and CSF from the cisterna magna were obtained from dogs, anesthetized with pentobarbital, preoperatively and at 3-week intervals thereafter. Heparinaized blood was immediately cooled on ice and centrifuged, and plasma was either processed immediately or kept frozen at −70°C. The plasma amino acid concentrations were determined by using a Beckman 121 MB amino acid analyzer with automatic integration. The plasma was deproteinized by mixing 0.5 ml plasma with 1.5 ml of 5% sulfosalicylic acid (pH 1.8, adjusted with LiOH) containing as an internal standard 133 nmol/ml thienylalanine. CSF samples were prepared by mixing 0.9 ml CSF with 0.1 ml 37.5%

sulfosalicylic acid, pH 2.0, containing 500 nmol/ml thienylalanine. After that, all samples were centrifuged (20,000 × g, 20 min, 4°C) and passed through a 0.4 um filter (Millipore Corp.) and frozen at −70°C until analysis.

At the time of sacrifice, the dogs were anesthetized with pentobarbital and blood and CSF samples were taken. The vault of the skull was removed by an electric saw and the brain disconnected at the junction with the spinal cord and rapidly transferred to ice-cold normal saline. The brain was then put on its dorsal surface on an ice-cold, saline-soaked paper towel. Nine regions of the brain were isolated separately and in the following order: hypothalamus, diencephalon, cerebellum, medulla, pons, mesencephalon, cortex, caudate nucleus, and hippocampus. The brain samples were immediately frozen in liquid nitrogen and stored at −70°C until the analysis. On the average, the samples were frozen within 5 min after the brain was removed. Hypothalamus was dissected by making a cut at a depth of approximately 5 mm from just anterior to the optic chiasma to the posterior aspect of the mamillary body bounded laterally by the choroid fissure. A sample was next taken from the diencephalon just dorsal to the hypothalamus. Cerebellum was then removed and a portion (1–2 g) taken for analysis. Pons was separated from the medulla at the pontine fissure. Mesencephalon was removed from the rest of the brain stem by a vertical cut just anterior to the superior colliculus. Next, the brain was divided into the two hemispheres and both caudate nuclei were removed from the lateral walls of the lateral ventricles. Hippocampal tissue was removed from the temporal recessus of the lateral walls of the lateral ventricles and cortical samples were taken from the temporoparietal areas.

The brain samples were homogenized in three volumes of 5% sulfosalicylic acid, pH 1.4, containing 133 nmol/ml of thienylalanine as internal standard. The samples were then handled in the same way as for plasma and CSF with the exception that each sample was analyzed twice, i.e., once at the 1:4 dilution of the homogenate for the low concentration amino acids, and then after further 1:25 dilution to measure the high concentration amino acids glutamate and glutamine. Plasma, brain and CSF were analyzed for threonine: (THR), glutamine (GLN), valine (VAL), methionine (MET), isoleucine (ILE), leucine (LEU), tyrosine (TYR), phenylalanine (PHE), tryptophan (TRP), histidine (HIS), and (in brain only) alanine (ALA) and glutamate (GLU).

Since one aim of this study is to determine whether the rise in the concentrations of various LNAA in the CNS could be accounted for solely by changes in circulating LNAA levels, the manner in which the circulating amino acid levels should be expressed is of importance. Studies in rats have demonstrated that the LNAA (THR, VAL, MET, ILE, LEU, TYR, PHE, HIS, TRP) compete for a common blood-brain transport system[17,25]. Glutamine can apparently be transported by this system also, but has a very low affinity for it[17,19]. Each LNAA has a characteristic affinity for the transport system, with some amino acids (e.g., LEU, PHE) competing more effectively for transport than others (e.g., VAL, THR). Studies from this laboratory in dogs have clearly demonstrated the effects of competition among plasma amino acids on CSF levels of LNAA [24]. Studies in dogs by others have suggested that the rank-order of transport Km's (affinities) is similar to that in rats [25]; however, no blood-brain transport Km values are available for normal dogs or in dogs after PCS. We expressed the availability of a particular amino acid for blood-brain transport in a form of the Michaelis-Menten kinetic formula, which accounted for the competition among plasma amino acids due both to their different concentrations in plasma and also to their presumed relative affinities (Km). The plasma competitor ratios (PCR) of the studied amino acids were calculated in a way similar to the brain influx rate used by Fernstrom and Faller [6] with the modification that the Vmax of transport was considered to be 100% as previously described [8]. One interpretation of uptake expressed in this fashion is that the calculated values represent the fraction of maximum transport activity expended on a particular amino acid. It is assumed, in making these calculations, that blood-brain LNAA transport does not greatly differ in dogs and rats and that PCA does not alter the affinities (K), of the amino acids for the transport system. The PCR were calculated as follows:

$$\mathrm{PCRx} = \frac{\mathrm{AAx}}{\mathrm{Kmx}[1 + \mathrm{AA}/\mathrm{Km}] + \mathrm{AAx}} \times 100\%$$

where AAx is the plasma concentration of the NAAx; Kmx is the transport Km of AAx; Km is the transport Km of competing NAA; AA is the plasma concentrations of competing NAA; and Vmax is the transport Vmax – 100%. The Km's of transport for each neutral amino acid from Pardridge and Mietus [19] and Pardridge and Oldendorf [20] were as follows: THR = 730 nmol/ml; VAL = 510 nmol/ml; ILE = 250 nmol/ml; LEU = 100 nmol/ml; MET = 180 nmol/ml; TYR = 150 nmol/ml; PHE = 110 nmol/ml; and HIS = 240 nmol/ml. Tryptophan concentrations were not included in the calculation since the actual amount of "free' or non-albumin-bound tryptophan was not determined. This fraction of the plasma tryptophan is relatively small and therefore exerts only slight competitive effect.

Statistical analysis of differences among serial samples of plasma and CSF was by analysis of variance and Newman-Keuls test. Correlation was tested by Pearson product-moment correlation. Comparison of amino acid concentrations in brain regions of control dogs and dogs killed in various stages of encephalopathy is by the non-parametric Kruskal-Wallis test.

### RESULTS

The interval from PCA until development of encephalopathy and sacrifice was highly variable, ranging from 21 to 213 days. The mean overall weight loss at time of sacrifice was $26 \pm 3\%$. The percentage weight loss was proportional to the grade of encephalopathy at time of sacrifice: $20 \pm 4\%$ in grade I, $29 \pm 4\%$ in grade II, and $34 \pm 4\%$ in grade III. At time of sacrifice six dogs were rated in grade I, 3 dogs in grade II, and 3 dogs in grade III. It should be noted that, due to the unpredictability of the course of encephalopathy after PCA in dogs, it proved difficult to achieve the goal of equal numbers of dogs in each stage of encephalopathy. In practice, those dogs which deteriorated quickly to stage II and III were sacrificed first, whereas those dogs which showed little tendency to develop severe encephalopathy were sacrificed at later times (up to several months) after surgery and were mostly in stage I.

Table 1 compares the changes in LNAA concentrations or competitor ratios at intervals after PCA with observed LNAA concentrations in cisternal CSF. The changes in the plasma amino acid pattern after PCA were similar to those previously reported [24] and characterized by increased concentrations of histidine and of the aromatic amino acids tyrosine and phenylalanine. The calculated plasma competitor ratios (Table 1) also changed after PCA in directions similar to the absolute plasma amino acid concentrations. Note that preoperatively the percentage of calculated transport activity was greatest for LEU and PHE, reflecting their high affinities for transport (low Km). After PCA the calculated ratios for PHE and TYR approximately doubled, representing the greatest increase of any of the LNAA in this parameter. The calculated competitor ratios for the BCAA decreased to a greater extent than their absolute concentrations in plasma.

**Table 1** Changes in plasma amino acid concentrations, calculated plasma competitor ratios, and cisternal CSF concentrations at various times after PCA.

| | Plasma (nmol/ml ± SEM) | | | | Plasma Competitor Ratio (% $V_{max}$ ± SEM) | | | | CSF (nmol/ml ± SEM) | | | |
|---|---|---|---|---|---|---|---|---|---|---|---|---|
| Amino Acid | pre-op | 3 wk | 6 wk | 9 wk | pre-op | 3 wk | 6 wk | 9 wk | pre-op | 3 wk | 6 wk | 9 wk |
| GLN | 671±26 | 1005±60<br>(+50%)[a] | 1068±80<br>(+59%)[a] | 961±112<br>(+43%)[a] | NC | | | | 447±13 | 1486±113<br>(+232%)[a] | 2034±264<br>(+355%)[a] | 1918±258<br>(+329%)[a] |
| THR | 203±29 | 167±16<br>(-18%) | 187±21<br>(-8%) | 162±25<br>(-20%) | 6.3±0.7 | 5.0±0.4<br>(-21%) | 5.4±0.6<br>(-14%) | 5.0±0.9<br>(-21%) | 49±5 | 42±5<br>(-14%) | 47±5<br>(-4%) | 44±5<br>(-10%) |
| VAL | 143±13 | 114±14<br>(-20%) | 113±16<br>(-21%) | 117±16<br>(-18%) | 7.5±0.2 | 4.8±0.3<br>(-36%) | 4.6±0.3<br>(-39%) | 4.9±0.5<br>(-35%) | 11±1 | 12±1<br>(+9%) | 14±1<br>(+27%)[a] | 16±2<br>(+45%)[a] |
| ILE | 37±6 | 37±5<br>(0) | 34±5<br>(-8%) | 37±2<br>(0) | 3.4±0.2 | 3.0±0.3<br>(-12%) | 2.3±0.2<br>(-33%) | 2.7±0.4<br>(-21%) | 4±1 | 4±1<br>(0) | 5±1<br>(+25%) | 5±1<br>(+25%) |
| LEU | 91±15 | 82±10<br>(-10%) | 78±11<br>(-14%) | 79±10<br>(-13%) | 22.7±0.9 | 17.9±1.2<br>(-21%) | 15.7±0.9<br>(-31%) | 17.1±1.7<br>(-25%) | 13±1 | 14±1<br>(+8%) | 17±1<br>(+31%) | 19±3<br>(+46%)[a] |
| MET | 45±3 | 48±4<br>(+7%) | 59±9<br>(+31%) | 42±6<br>(-7%) | 6.9±0.4 | 5.8±0.3<br>(-16%) | 7.1±0.7<br>(+3%) | 5.8±0.5<br>(-16%) | 8±1 | 18±3<br>(+125%)[a] | 23±3<br>(+187%)[a] | 21±3<br>(+162%)[a] |
| TYR | 32±4 | 75±7<br>(+134%)[a] | 98±10<br>(+206%)[a] | 82±9<br>(+156%)[a] | 5.7±0.4 | 10.8±0.4<br>(+89%) | 12.1±0.3<br>(+112%) | 11.3±0.8<br>(+98%) | 9±1 | 32±3<br>(+256%)[a] | 53±6<br>(+489%)[a] | 42±7<br>(+367%)[a] |
| PHE | 43±4 | 102±10<br>(+137%)[a] | 132±20<br>(+207%)[a] | 109±10<br>(+153%)[a] | 10.3±0.5 | 20.1±0.7<br>(+95%) | 21.2±1.5<br>(106%) | 20.6±1.9<br>(+100%) | 12±1 | 43±3<br>(+258%)[a] | 72±8<br>(+500%)[a] | 61±14<br>(+408%)[a] |
| HIS | 64±3 | 93±7<br>(+45%)[a] | 117±11<br>(+83%)[a] | 99±6<br>(+55%)[a] | 7.4±0.4 | 8.6±0.4<br>(+16%) | 9.7±0.4<br>(+31%) | 9.0±0.9<br>(+22%) | 13±1 | 34±3<br>(+162%)[a] | 49±3<br>(+277%)[a] | 44±10<br>(+238%)[a] |
| TRP | 59±4 | 66±9<br>(+12%) | 83±10<br>(41%) | 60±9<br>(+2%) | NC | | | | 5±1 | 14±1<br>(+180%)[a] | 18±2<br>(+260%)[a] | 17±3<br>(+240%)[a] |

Percent change from pre-op indicated beneath values.
NC = not calculated (see text).
Number of dogs = pre-op, 12; 3 wk, 12; 6 wk, 9; 9 wk, 5.
[a] Significantly different from controls; $p<0.01$

The CSF concentration of GLN, presumably the product of cerebral ammonia detoxification with glutamic acid, rose after PCA to a much greater degree than in plasma. The increases in the concentration of PHE, TYR, HIS and TRP in CSF greatly exceeded the change either in absolute plasma levels or in the calculated competitor ratios. In all brain regions of dogs with PCA, the concentrations of GLN, TYR, PHE, TRP and HIS were several-fold higher than control, while MET and LEU were increased to a lesser extent. The regional concentrations of alanine (ALA) and of glutamic acid (GLU), the precursor for GLN synthesis, are also shown in Table 2 for comparison, although they are not substrates for the LNAA transport system of the blood-brain barrier. There was no consistent statistical difference in regional amino acid levels between dogs sacrificed in encephalopathy grade I as compared with dogs in grades II and III combined. Combining the data of dogs in grade II and III was necessary to achieve sufficient numbers for statistical analysis.

**Table 2** Regional concentrations of amino acids in the brain of dogs before and after portacaval anastomosis.

| Brain Region | Grade of Encephalopathy | Amino Acid THR | GLU | GLN | ALA | VAL | MET | ILE | LEU | TYR | PHE | TRP | HIS |
|---|---|---|---|---|---|---|---|---|---|---|---|---|---|
| Diencephalon | Controls (4) | 262 | 9039 | 4670 | 371 | 98 | 42 | 25 | 57 | 42 | 35 | 18 | 160 |
| | I (6) | 154[a] | 8125 | 22320[a] | 381 | 95 | 92[a] | 29 | 88[a] | 235[a] | 244[a] | 65[a] | 544[a] |
| | II + III (6) | 157[a] | 8654 | 18184[a] | 378 | 122 | 62[a] | 35[a] | 123[a] | 221[a] | 308[a] | 77[a] | 410[a] |
| Hypothalamus | Controls | 214 | 4359 | 3498 | 262 | 72 | 29 | 15 | 51 | 37 | 35 | 23 | 61 |
| | I | 144 | 4861 | 13577[a] | 279 | 70 | 65[a] | 15 | 49 | 158[a] | 166[a] | 49[a] | 206[a] |
| | II + III | 132[a] | 5089 | 16463[a] | 425 | 87 | 62[a] | 21 | 77[a] | 144[a] | 196[a] | 52[a] | 194[a] |
| Medulla | Controls | 263 | 5809 | 3468 | 421 | 107 | 44 | 35 | 63 | 51 | 50 | 22 | 133 |
| | I | 165[a] | 6048 | 17941[a] | 428 | 111 | 63[a] | 38 | 84 | 247[a] | 291[a] | 63[a] | 420[a] |
| | II + III | 124[a] | 5667 | 18847[a] | 365 | 136 | 86[a] | 33 | 103 | 210[a] | 321[a] | 68[a] | 368[a] |
| Pons | Controls | 278 | 5952 | 2984 | 406 | 83 | 37 | 22 | 60 | 48 | 50 | 22 | 115 |
| | I | 192 | 4786 | 11734[a] | 375 | 86 | 105[a] | 19 | 73 | 246[a] | 322[a] | 63[a] | 376[a] |
| | II + III | 141[a] | 5191 | 19149[ab] | 458 | 90 | 86[a] | 27 | 100[a] | 319[a] | 321[a] | 68[a] | 199[a] |
| Cortex | Controls | 281 | 10654 | 6174 | 590 | 126 | 61 | 33 | 86 | 55 | 52 | 20 | 108 |
| | I | 145[a] | 9460 | 23506[a] | 605 | 231 | 89[a] | 34 | 100 | 260[a] | 295[a] | 76[a] | 410[a] |
| | II + III | 128[a] | 9541 | 32773[a] | 596 | 238 | 69[a] | 36 | 134[a] | 247[a] | 266[a] | 76[a] | 303[a] |
| Caudate Nucleus | Controls | 356 | 11510 | 7031 | 481 | 93 | 45 | 24 | 72 | 48 | 47 | 24 | 103 |
| | I | 184[a] | 10729 | 23709[a] | 548 | 73 | 101[a] | 27 | 89 | 234[a] | 230[a] | 67[a] | 349[a] |
| | II + III | 141[a] | 10937 | 21287[a] | 906 | 115 | 71[a] | 42 | 121 | 206[a] | 280[a] | 73[a] | 294[a] |
| Hippocampus | Controls | 362 | 8451 | 4492 | 733 | 98 | 52 | 24 | 71 | 51 | 41 | 20 | 172 |
| | I | 189[a] | 8273 | 16954[a] | 753 | 88 | 83[a] | 23 | 84 | 234[a] | 231[a] | 67[a] | 544[a] |
| | II + III | 122[a] | 9115 | 16894[a] | 755 | 116 | 65[a] | 36 | 111 | 203[a] | 273[a] | 63[a] | 509[a] |
| Mesencephalon | Controls | 291 | 6239 | 3478 | 498 | 153 | 41 | 22 | 63 | 48 | 45 | 21 | 140 |
| | I | 178[a] | 6217 | 18516[a] | 420 | 115 | 99[a] | 32 | 85 | 248[a] | 268[a] | 63[a] | 429[a] |
| | II + III | 128[a] | 6637 | 19125[a] | 474 | 130 | 72[a] | 35 | 107[a] | 223[a] | 296[a] | 65[a] | 444[a] |
| Cerebellum | Controls | 345 | 8561 | 6566 | 370 | 112 | 51 | 27 | 95 | 59 | 61 | 27 | 120 |
| | I | 187[a] | 8753 | 17748[a] | 415 | 113 | 112[a] | 20 | 90 | 237[a] | 293[a] | 67[a] | 288[a] |
| | II + III | 154[a] | 8706 | 23030[a] | 513 | 134 | 79 | 22 | 131 | 232[a] | 351[a] | 61[a] | 292[a] |

Results are given as the median since a non-parametric test was used for the calculations.

[a]Significantly different from the controls; $p<0.05$ at least.

[b]Significant difference between encephalopathy grade I vs. grade II + III; $p<0.05$ at least.

We tested whether the concentration of each of the LNAA in CSF was more closely correlated to the plasma competitor ratio of that amino acid or to the CSF concentration of GLU (Table 3). For these correlations, 45 data-pairs were considered, significant correlations were observed between the CSF concentrations of THR, TYR, PHE and HIS and their respective plasma competitor ratios. Significant correlations were also found between the CSF concentration of GLN and that of MET, LEU, TYR, PHE and HIS. For all CSF amino acids except THR, the correlation with CSF GLN was better (higher value of the correlation coefficient (r) than with the plasma competitor ratio, suggesting that CSF LNAA concentrations are more strongly influenced by the CNS concentration of GLN than by the availability of LNAA for transport from the circulation. The relationship between CSF GLN and the four LNAA with the best correlations are shown graphically in Figure 1.

**Table 3** Pearson product-moment correlations between CSF concentrations of LNAA and either the plasma competitor ratio (PCR) or CSF GLN.

| Amino Acid | versus PCR (r) | versus CSF GLN (r) |
|---|---|---|
| THR | 0.433[a] | 0.033 |
| VAL | 0.170 | 0.340 |
| MET | -0.138 | 0.654[b] |
| ILE | 0.057 | 0.144 |
| LEU | 0.035 | 0.527[b] |
| TYR | 0.720[b] | 0.950[b] |
| PHE | 0.790[b] | 0.914[b] |
| HIS | 0.439[a] | 0.904[b] |

[a] $p<0.01$

[b] $p<0.001$

A similar comparisons of correlations was performed between the regional brain concentration of each LNAA and either the plasma competitor ratio or either concentration of GLN in that region (Table 4). For these correlations, all 16 dogs were considered. As in the CSF, the best correlations were observed for MET, PHE, TYR and HIS. For MET and HIS, the better correlations were clearly with the brain GLN concentration; whereas, for PHE and TYR, their brain concentrations correlated about equally well both with the plasma competitor ratio and the brain GLN level.

The correlation between CSF GLN and regional GLN was tested in the twelve dogs with a PCS. The controls were omitted in this case since including them would have markedly improved all the correlations, statistically significant correlation was obtained only for cortex ($r = 0.645$, $p<0.05$. Figure 2) and for cerebellum ($r = 0.593$, $p<0.05$). This result may reflect the fact that cerebral cortex and cerebellum constitute two of the largest brain regions in dogs and, hence, diffusion of amino acids from these regions may have considerable influence on the composition of CSF.

### DISCUSSION

The lack of correlation in the present studies between encephalopathy grade and brain levels of amino acids, which are known or thought to be involved in synthesis

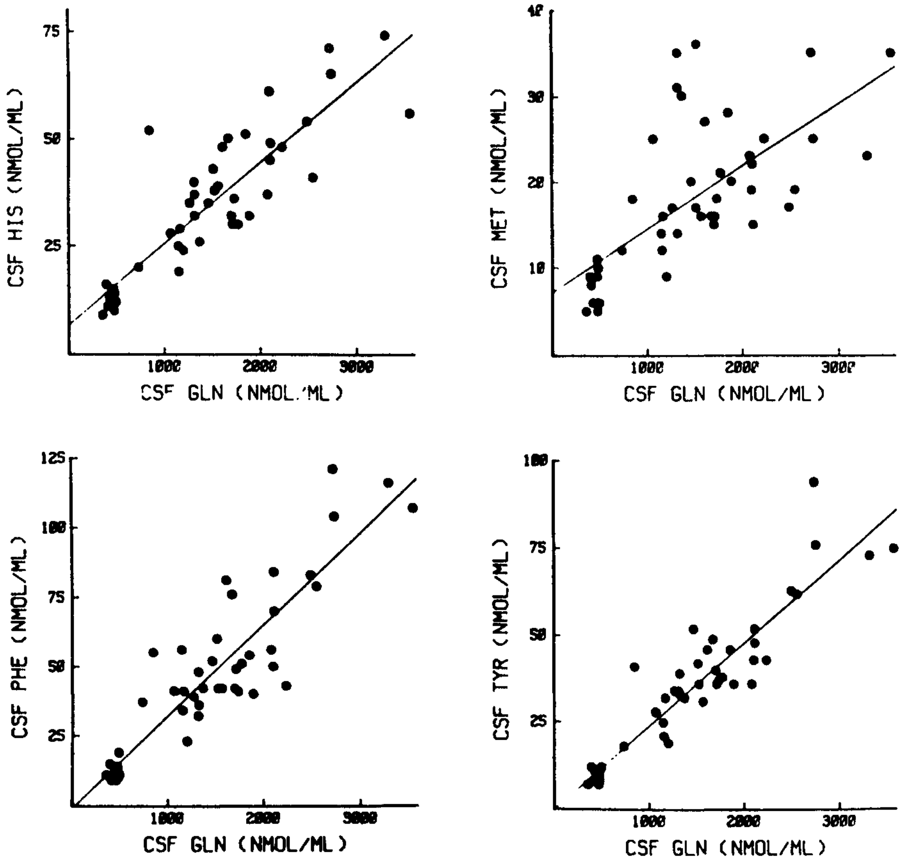

**Figure 1** Relationship between the CSF concentrations of GLN and CSF concentrations of HIS, MET, PHE and TYR. The data points shown represent pre-operative samples, clustered at lower left of each panel, and all subsequent CSF samples obtained on all dogs. Correlation coefficients are shown in Table 3.

of true or false neurotransmitters, would seem to weaken the hypothesis that changes in brain amino acid levels are involved in encephalopathy. However, it should be noted that the design of the experiment may have resulted in significant bias if there was some difference in "sensitivity" to high brain LNAA levels in those dogs which exhibited encephalopathy at early or late times after the PCA. We have no evidence, however, that such differences in sensitivity exist.

Models of blood-brain amino acid transport consider the existence of at least three compartments: (a) blood or plasma, (b) brain extracellular fluid and the CSF, and (c) the intracellular fluid of brain cells[22]. Amino acids in a cerebral capillary must be transported across the two plasma membranes of the capillary endothelial cell in order to enter the extracellular fluid where they are then available to the transport systems of the brain cells themselves. Similarly, to exit from brain, amino acids must cross the brain cell membrane into the extracellular fluid and then must cross the two capillary membranes in order to reach the circulation. The total

**Table 4** Correlation of regional brain concentrations of several LNAA with the plasma competitor ratio and with the regional brain GLN concentration.

| METHIONINE Region | PCR vs Brain Concentration (r) | Brain GLN vs Brain Concentration (r) |
|---|---|---|
| Dien | 0.042 | 0.590[a] |
| Hypo | 0.254 | 0.482 |
| Med | -0.168 | 0.618[b] |
| Pons | -0.147 | 0.369 |
| Cortex | 0.185 | 0.536[a] |
| CN | 0.173 | 0.513[a] |
| Hippo | -0.135 | 0.461 |
| Mesen | 0.002 | 0.601[a] |
| Cereb | 0.208 | 0.676[b] |

| TYROSINE Region | PCR vs Brain Concentration (r) | Brain GLN vs Brain Concentration (r) |
|---|---|---|
| Dien | 0.901[c] | 0.950[c] |
| Hypo | 0.803[c] | 0.924[c] |
| Med | 0.852[c] | 0.927[c] |
| Pons | 0.767[c] | 0.694[c] |
| Cortex | 0.929[c] | 0.855[c] |
| CN | 0.904[c] | 0.902[c] |
| Hippo | 0.899[c] | 9.929[c] |
| Mesen | 0.886[c] | 0.914[c] |
| Cereb | 0.857[c] | 0.928[c] |

| PHENYLALANINE Region | PCR vs Brain Concentration (r) | Brain GLN vs Brain Concentration (r) |
|---|---|---|
| Dien | 0.951[c] | 0.948[c] |
| Hypo | 0.889[c] | 0.952[c] |
| Med | 0.943[c] | 0.980[c] |
| Pons | 0.903[c] | 0.673[b] |
| Cortex | 0.954[c] | 0.842[c] |
| CN | 0.972[c] | 0.957[c] |
| Hippo | 0.961[c] | 0.964[c] |
| Mesen | 0.945[c] | 0.946[c] |
| Cereb | 0.924[c] | 0.960[c] |

| HISTIDINE Region | PCR vs Brain Concentration (r) | Brain GLN vs Brain Concentration (r) |
|---|---|---|
| Dien | 0.670[b] | 0.861[c] |
| Hypo | 0.467 | 0.891[c] |
| Med | 0.567[a] | 0.938[c] |
| Pons | 0.703[b] | 0.395 |
| Cortex | 0.590[a] | 0.680[b] |
| CN | 0.534[a] | 0.924[c] |
| Hippo | 0.563[a] | 9.941[c] |
| Mesen | 0.540[a] | 0.933[c] |
| Cereb | 0.540[a] | 0.953[c] |

[a] $p<0.05$
[b] $p<0.01$
[c] $p<0.001$

capillary surface area is very small compared to the sum of brain cell membranes. It has been proposed that the limiting factor in blood-brain transport of amino acids (as well as of other substances requiring carrier-mediated transport) is transport across the capillary membranes[21].

In the present studies, with the exception of THR, the magnitude of the increase or decrease in absolute plasma LNAA levels was not the same as the changes in concentration of amino acids in CSF or in brain. The magnitude of change in the calculated plasma competitor ratios for each LNAA was also considerably less than the change in CSF or brain LNAA after PCA. These observations strongly suggest that some factor other than the change in plasma LNAA concentrations was responsible for the rise in CSF and brain LNAA levels after PCA.

Elevated concentrations of GLN in lumbar CSF are commonly found in patients with encephalopathy resulting from liver disease[9]. The synthesis in brain of GLN from ammonia and glutamic acid apparently occurs very rapidly[4] and primarily in glial astrocytes, where the enzyme glutamine synthetase is located[16]. The elevated CSF GLN concentration in patients almost certainly reflects accelerated detoxification of ammonia in brain secondary to hyperammonemia[14]. In the present studies, the CSF GLN concentration rose from levels which were less than those in plasma preoperatively to levels which were markedly greater than in plasma (Table 1).

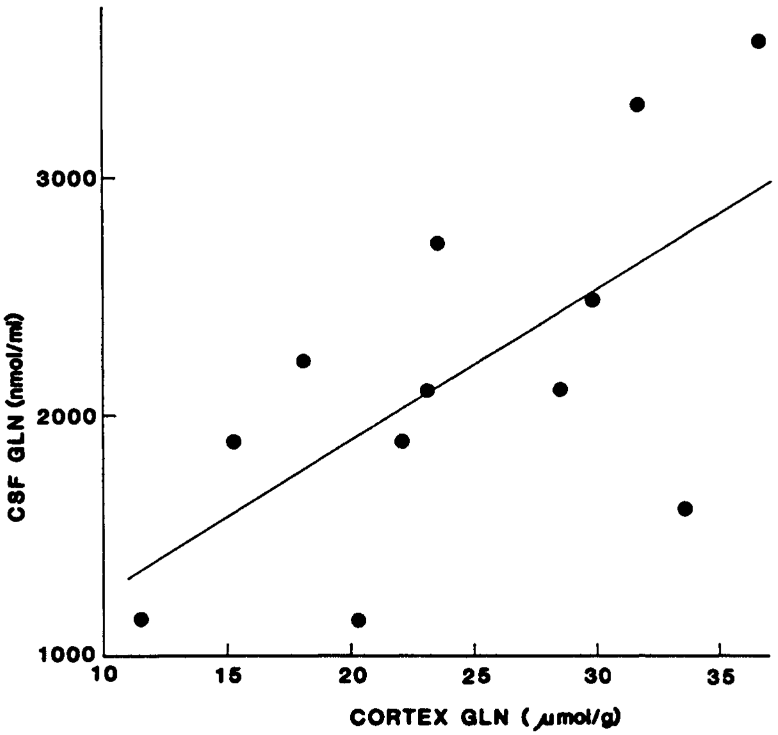

**Figure 2** The relationship of cisternal CSF GLN to GLN in cortex in the twelve dogs with PCA at time of sacrifice ($r = 0.645$, $p < 0.05$).

This change in the CSF/plasma GLN ratio is strong evidence that transport of GLN from extracellular fluid to blood across the capillary barrier was slow compared to the rate of GLN synthesis, at least until CSF GLN reached a much higher level. Presumably, at the higher level, synthesis of GLN was matched by the rate of removal of GLN from the ECF. The present studies suggest that competition for transport from ECF to blood among GLN and the other LNAA can be an important determinant of brain LNAA levels in liver disease.

The increased CSF concentration of several of the LNAA was better correlated with the increased CSF GLN concentration than with the change in the plasma competitor ratios of those amino acids (Table 3). This does not imply that competition among plasma neutral amino acids for transport across the blood-brain barrier is not an important determinant of brain amino acid levels. On the contrary, Smith, *et al.* [24] showed that infusing a solution rich in the BCAA could drastically reduce the CSF levels of the other LNAA in dogs with portal-systemic encephalopathy. The lower degree of correlation between plasma competitor ratios and CSF LNAA levels suggests that, under the pathological conditions of the present study, the GLN concentration in the CNS was a more important determinant of the concentrations of the LNAA in the brain or CSF.

The most direct means by which a high concentration of GLN in the brain ECF might affect the levels of other LNAA in brain ECF is for GLN to compete with the other LNAA for transport out of brain. If transport of the LNAA across the capillary endothelial barrier in the blood-to-brain direction is, as is thought, primarily determined by competition, then it is very likely that transport in the brain-to-blood direction is primarily determined by competition also. Increased concentrations of GLN in CSF would inhibit the efflux of other LNAA until sufficient concentrations of the other LNAA had accumulated in the ECF to overcome this competition for transport out of brain. At this new steady state the relationship between influx and efflux of the LNAA would be restored to more normal levels. Accordingly to this hypothesis, the rise in LNAA concentrations in the ECF would be a necessary and passive consequence of the increase in GLN concentration in the same compartment.

The rise in brain intracellular concentrations of the LNAA may be explained by assuming that the amino acid transport systems of brain cells include concentrative mechanisms which actively "pump" amino acids into brain cells against their concentration gradients[23]. If it is further assumed that these transport systems are capable of establishing a gradient with a fixed inside/outside concentration ratio of 3:1 to 4:1, then the rise in brain LNAA concentrations would follow as a simple consequence of the rise in ECF LNAA concentrations. In the present studies, the observation that the changes in CSF and in brain tissue amino acids were of similar magnitude after PCA is consistent with this assumption.

It should be recalled that the metabolic fate of the various LNAA will affect their final concentrations intracellularly. None of the LNAA, except GLN and perhaps a small amount of TYR (from pHE), can be synthesized in brain. The brain can metabolize the leucine and probably the other BCAA [3]. It is therefore likely that the small change in brain levels of the BCAA after PCA, as compared with the greater increase in the AAA, is due in part to more rapid metabolism of the BCAA. It is nonetheless notable that brain and CSF LEU concentrations rose after PCA even though plasma LEU fell.

This hypothetical sequence of events does not require that the rate of transport of LNAA across the blood-brain barrier actually be increased in the presence of hyperammonemia and portal-systemic shunting. Changes in the rate of such transport have been observed in rats after PCA[11,26,15], but attempts to demonstrate accelerated blood-brain transport in dogs after PCA have not been successful[10]. If the unidirectional flux of LNAA from blood to brain was, in fact, increased in dogs with PCA, then the principal effect of this would be to accelerate the accomplishment of the new steady state levels of LNAA inthe ECF, which in turn is determined by the ECF GLN concentration.

These various factors affecting LNAA levels after PCA in CSF or in brain tissue are summarized schematically in Figure 3. Compared to normal (A), the only change shown immediately after PCA (B) is a rise in blood ammonia. In actuality the changes in blood ammonia and in plasma LNAA concentrations probably proceed simultaneously, but for the purpose of discussion we will consider these two processes as distinct. As a consequence of hyperammonemia, the synthesis of glutamine in astrocytes is stimulated, resulting in increased brain GLN concentrations. Panel (B) also represents a hypothetical but possibly observable moment when brain GLN is elevated, yet the CSF GLN level has not yet begun to rise. According to the role of GLN in blood-brain LNAA transport proposed previously in this discussion, no change in CSF or brain LNAA levels should occur before CSF GLN becomes elevated.

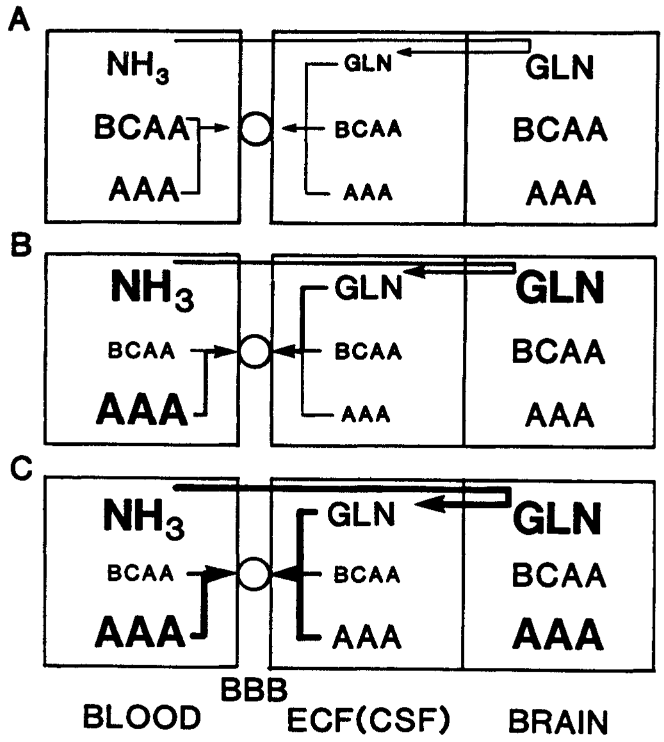

**Figure 3** Proposed schema reflecting events which occur after PCA in blood, CSF or extracellular fluid (ECF) and brain compartments with respect to ammonia ($NH_3$), BCAA and AAA. Panel A shows normal pre-operative levels of ammonia and amino acids in blood plasma, CSF and brain tissue. Smaller type-face in ECF reflects much lower concentrations in that compartment. Panel B represents a hypothetical period after creation of the PCA when blood ammonia is elevated, thus raising the brain GLN concentration, but before the CSF concentrations of GLN has risen and thus before blood-brain NAA transport has been affected.
In Panel C, plasma levels of AAA are elevated while those of BCAA are decreased, thus altering plasma competitor ratios. High GLN in the ECF plus altered competition for transport result in greatly increased brain AAA concentrations.

In panel (C) the characteristic changes in plasma LNAA after PCA alter the plasma competitor ratios and thus the ratio of AAA/BCAA which can be transported into brain. The elevated ECF GLN now competes for brain-to-blood transport with the other LNAA, resulting in higher ECF and thus brain levels of the AAA. Brain levels of BCAA are little affected both because the influx of BCAA is somewhat reduced and because these amino acids are metabolized in brain.

### *References*

1. Cangiano, C., Cardelli-Cangiano, P., James, J.H., Rossi-Fanelli, F., Patrizi, M.A., Brackett, K.A., Strom, R., and Fischer, J.E. (1983) Brain microvessels take up large neutral amino acids in exchange for glutamine. *J. Biol. Chem.*, **258**, 8949–8954

2. Cascino, A., Cangiano, C,. Fiaccadori, F., Ghinelli, F., Merli, M., Pelosi, G., Riggio, O., Rossi-Fanelli, F., Sacchini, D., Stortoni, M., and Capocaccia, L. (1982) Plasma and cerebrospinal fluid amino acid patterns in hepatic encephalopathy. *Dig. Dis. Sci.* **27**, 828–832
3. Chaplin, E.R., Goldberg, A.L., and Diamond, I. (1976) Leucine oxidation in brain slices and nerve endings. *J. Neurochem.*, **26**, 701–707
4. Cooper, A.J.L., McDonald, J.M., Gelbard, A.S., Gledhill, R.F., and Duffy,, T.E. (1979) The metabolic fate of 13N-labeled ammonia in rat brain. *J. Biol. Chem.*, **254**, 4982–4992
5. Cummings, M.G., Soeters, P.B., James, J.H., Neane, J.M., and Fischer, J.E. (1976) Regional brain indoleamine metabolism following chronic portacaval anastomosis in the rat. *J. Neurochem.*, **27**, 501–508
6. Fernstrom, J.D., and Faller, D.V. (1978) Neutral amino acids in the brain; Changes in response to food ingestion. *J. Neurochem.*, **30**, 1531–1538
7. Fischer, J.E., and Baldessarini, R.J. (1971) False neurotransmitters and hepatic failure. *Lancet*, **10**, 75–80
8. Herlin, P.M., James, J.H., Joffe, S.N., Kulneff-Herlin, A.E.A., and Fischer, J.E. (1982) Effect of jejunoileal bypass on plasma and brain amino acids in the rat. *J. Neurochem.*, **38**, 1170–1173
9. Hourani, B.T., Hamlin, E.M., and Reynolds, T.B. (1971) Cerebrospinal fluid glutamine as a measure of hepatic encephalopathy. *Arch. Intern. Med.*, **127**, 1033–1036
10. Huet, P-M., Pomier-Layrargues, G., Duguay, L., and duSoich, P. (1981) Blood-brain transport of tryptophan and phenylalanine: Effect of portacaval shunt in dogs. *Am. J. Physiol.*, **241**, G169–G173
11. James, J.H., Escourrou, J., and Fischer, J.E. (1978) Blood-brain neutral amino acid transport activity is increased after portacaval anastomosis. *Science*, **200**, 1395–1397
12 James, J.H., Jeppsson, B., Ziparo, V. and Fischer, J.E. (1979) Hyper- anmonaemia, plasma amino acid imbalance, and blood-brain amino acid transport: A unified theory of portal-systemic encephalopathy. *Lancet*, **13**, 722–775
13. Rigotti, P., Jonung, T., Peters, J.C., James, J.H., and Fischer, J.E. (1985) Methionine sulfoximine prevents the accumulation of large neutral amino acids in brain of portacaval-shunted rats. *J. Neurochem.*, **44**, 929–933
14. Lockwood, A.H., McDonald, J.M., Reiman, R.E., Gelbard, A.S., Laughlin, J.S., Duffy, T.E., and Plum, F. (1979) The dynamics of ammonia metabolism in man: Effects of liver disease and hyperammonemia. *J. Clin. Invest.*, **63**, 449–460
15. Mans, A.M., Biebuyck, J.F., Shelly, K., and Hawkins, R.A. (1982) Regional blood-brain barrier permeability to amino acids after portacaval anastomosis. *J. Neurochem.*, **38**, 705–717
16. Norenberg, M.D. and Martinez-Hernandez, A. (1979) Fine-structural localization of glutamine synthetase in astrocytes of rat brain. *Brain Res.*, **161**, 303–310
17. Oldendorf, W.H., and Szabo, J. (1976) Amino acid assignment to one of three blood-brain barrier amino acid carriers. *Am. J. Physiol.*, **230**, 94–98
18. Ono, J., Hutson, D.G., Dombro, R.S., Levi, J.U., Livingstone, A., and Zeppa, R. Tryptophan and hepatic coma. *Gastroenterol.*, **74**, 196–200
19. Pardridge, W.M., and Mietus, L.J. (1982) Kinetics of neutral amino acid transport through the blood-brain barrier of the newborn rabbit. *J. Neurochem.*, **38**, 955–962
20. Pardridge, W.M., and Oldendorf, W.H. (1975) Kinetic analysis of blood-brain barrier transport of amino acids. *Biochim. Biophys. Acta*, **401**, 128–136
21. Pardridge, W.M., and Oldendorf, W.H. (1977) Transport of metabolic substances through the blood-brain barrier. *J. Neurochem.*, **28**, 5–12
22. Rapaport, S.I. (1976) *Blood-Brain Barrier in Physiology and Medicine.* New York: Raven Press, p. 90
23. Sershen, H., and Lajtha, A. (1979) Inhibition pattern by analogs indicates the presence of ten or more transport systems for amino acids in brain cells. *J. Neurochem.* **32**, 719–726
24. Smith, A.R., Rossi-Fanelli, F., Ziparo, Y., James, J.H., Perelle, B.A., and Fischer, J.E., (1978) Alterations in plasma and CSF amino acids, amines and metabolites in hepatic coma. *Ann. Surg.*, **187**, 343–350
25. Yudilevich, D.L., De Rose, N., and Sepulveda,F.V. (1972) Facilitated transport of amino acids through the blood-brain barrier of the dog studied in a single capillary circulation. *Brain Res.*, **44**, 569–578
26. Zanchin, G., Rigotti, P., Dussini, N., Vassanelli, P., and Battistin, L. (1979) Cerebral amino acid levels and uptake in rats after portocaval anastomosis: II. Regional studies *in vivo*. *J. Neurosci. Res.*, **4**, 301–310

*Footnote*

In conducting this research, the investigators adhered to the "Guide for the Care and Use of Laboratory Animals" as promulgated by the Committee of Care and Use of Laboratory Animals in the Institute of Laboratory Animal Resources, National Research Council.

*(Accepted by S. Bengmark 23 April 1991)*

### INVITED COMMENTARY

The CNS findings in PSE animals are impressive and confirm previous studies showing a relation between the amino acid changes and CNS ammonia metabolism with CNS glutamine accumulation[1] and abnormal neurotransmission[2]. However, a major question remains untested. Are the changes demonstrated as a result of PSE or liver failure? The decrease in the ratio of BCAA/AAA has already been shown to occur in liver dysfunction in the absence of PSE[3], to decrease further with the development of cirrhosis[4] and to associated increase with an improvement in liver function on Prednisone therapy[5]. Furthermore, these changes may be only secondarily associated with liver dysfunction; closely associated with elevated insulin levels[6] and may in themselves affect hepatocyte protein synthesis and therefore plasma amino acid levels[7]. Thus it appears clear that the correct control group for the PCA animal model is an animal model of liver disease in the absence of PSE. For example, chronic bile duct ligation.

*References*

1. Giguere, J.F., Butterworth, R.F. (1984) Amino acid changes in regions of the CNS in relation to function in experimental portal-systemic encephalopathy. *Neurochemical Research*, **9**, 1309–1321
2. Ferenci, P., Riederer, P., Pappas, S.C., Jones, E.A. (1984) Effects of Branched Chain Amino Acids on Ammonia Induced Changes in Neurotransmission. Branched-Chain Amino and Keto Acids in Health and Disease. pp. 472–482 Basel: Karger
3. Morgan, M.Y., Milsom, J.P. and Sherlock, S. (1978) Plasma ratio of valiane, leucine and isoleucine to phenylalanine and tyrosine in liver disease. *Gut*, **19**, 1068–1073
4. McCullough, A.J., Czaja, J., Jones, J.D. and Go, V.L.W. (1981) The Nature and Prognostic Significance of Serial Amino Acid Determinations in Severe Chronic Active Liver Disease. *Gastroenterology*, **81**, 645–652
5. Ferenci, P., Bratusch-Marrain, P., Waldhausl, W.K., Nowotny, P. and Korn, A. (1984) Impaired plasma amino-acid clearance in patients with cirrhosis of the liver and portocaval shunt — its relation to insulin resistance. *European Journal of Clinical Investigation*, **14**, 255–261
6. Marchesini, G., Corlani, G., Zoli, M., Dondi, C., Bianchi, G., Bua, V., Vannini, P. and Pisi, E. (1983) Effect of Euglycemic Insulin Infusion on Plasma Levels of Branched-Chain Amino Acids in Cirrhosis. *Hepatology* **3**, 184–187
7. Montoya, A., Gomez-Lechon, M.J. and Castell, J.V. (1987) Influence of branched-chain amino acids on the synthesis of plasma proteins by cultured rat hepatocytes. *J. Clin. Nutr. Gastroenterol.*, **2**, 117–125

Laurence Blendis
Toronto General Hospital
9th Floor, Eaton Wing
Toronto, Ontario
Canada

